# Therapeutic Benefits of Tocilizumab Vary in Different Organs of a Patient with AA Amyloidosis

**DOI:** 10.1155/2014/823093

**Published:** 2014-08-12

**Authors:** Masaru Matsui, Satoshi Okayama, Hideo Tsushima, Kenichi Samejima, Tomoko Kanki, Ayako Hasegawa, Katsuhiko Morimoto, Yasuhiro Akai, Masato Takano, Shiro Uemura, Chiho Ohbayashi, Yoshihiko Saito

**Affiliations:** ^1^First Department of Internal Medicine, Nara Medical University, 840 Shijo-cho, Kashihara, Nara 634-8522, Japan; ^2^Department of Diagnostic Pathology, Nara Medical University, 840 Shijo-cho, Kashihara, Nara 634-8522, Japan

## Abstract

Systemic reactive AA amyloidosis is a life-threatening complication of chronic inflammatory diseases. Anti-interleukin-6 receptor, tocilizumab (TCZ), has been shown to improve clinical symptoms of patients with AA amyloidosis, accompanied with regression of the amyloid deposition. We report a case of AA amyloidosis evaluated by histology of multiple organs before and after TCZ treatment. A woman in her 60s with rheumatoid arthritis was referred to our hospital because of cardiac and renal dysfunction. A gastric and renal biopsy revealed the deposition of AA amyloid, and echocardiography revealed concentric left ventricular hypertrophy. Her estimated glomerular filtration rate was decreased to 8.6 mL/min/1.73 m^2^, and B-type natriuretic peptide, C-reactive protein, and serum amyloid A protein were significantly elevated. TCZ treatments markedly decreased her serum amyloid A protein and C-reactive protein levels, but hemodialysis was required 1 year later. Endoscopic gastric rebiopsy 3 years after initiation of TCZ treatments revealed the regression of amyloid deposition and echocardiography revealed improvement of her left ventricular hypertrophy. However, a renal rebiopsy revealed that the amyloid deposition had not regressed. In conclusion, these observations suggest that the therapeutic effects of TCZ can vary among organs in patients with AA amyloidosis.

## 1. Introduction

Systemic reactive AA amyloidosis is a life-threatening complication of chronic inflammatory diseases, such as rheumatoid arthritis (RA), latent tuberculosis, and bronchiectasis, and is characterized by the extracellular deposition of amyloid fibrils derived from serum amyloid A protein (SAA) [1]. SAA is synthesized in hepatocytes following stimulation by proinflammatory cytokines, such as interleukin- (IL-) 1, IL-6, and tumor necrosis factor-alpha, suggesting that these cytokines are potential therapeutic targets for the treatment of AA amyloidosis [2, 3].

Tocilizumab (TCZ) is a humanized monoclonal antibody that competitively inhibits the binding of IL-6 to its receptor [4]. TCZ has been shown to suppress the activity of RA and to improve clinical symptoms of AA amyloidosis secondary to RA, such as diarrhea, proteinuria, and cardiac hypertrophy [5–9]. However, few studies have reported the different therapeutic effects of TCZ on different organs or the comparative histology of multiple organs before and after TCZ treatment. Here, we describe a patient with AA amyloidosis who was treated with TCZ, after which her gastric manifestations improved; however, the renal amyloid deposition did not regress with treatment.

## 2. Case Presentation

**Figure 1:**
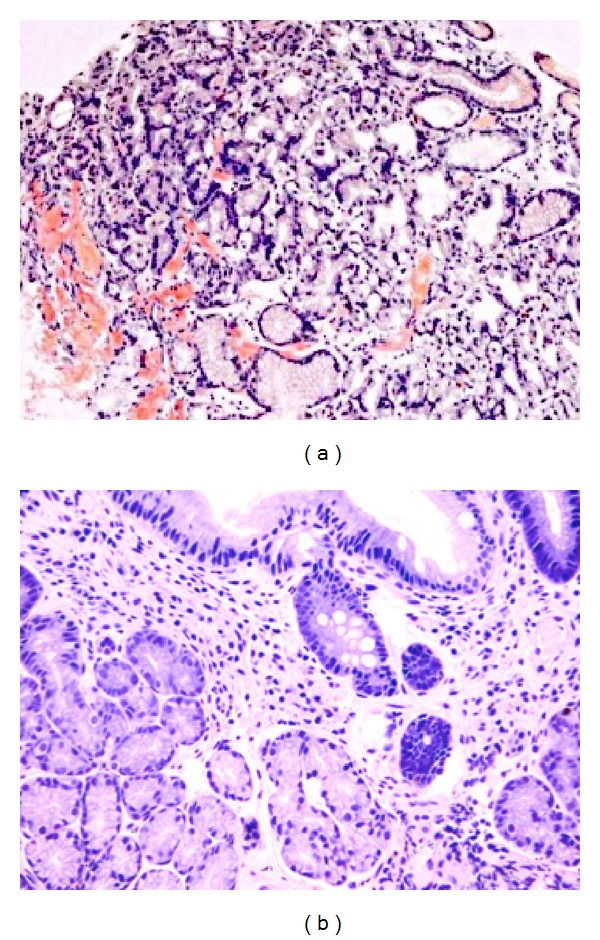
Endoscopic gastric biopsy. (a) Congo red staining shows amorphous amyloid deposits in the gastric mucosa. (b) After 4 years of tocilizumab treatments, regression of the amyloid deposition was noted.

A woman in 60s was referred to our hospital because of heart failure and renal dysfunction. She had suffered from RA for approximately 10 years, and its activity could not be sufficiently suppressed. She had been treated with prednisolone (7.5 mg/day), bucillamine (200 mg/day), and methotrexate (8 mg/week), but she continued to experience joint pain in addition to having high levels of C-reactive protein (CRP). She had been admitted to another hospital because of dyspnea 3 months before this presentation. Her renal function worsened, with her estimated glomerular filtration rate (eGFR) decreasing from 32.1 to 11.5 mL/min/1.73 m^2^. An endoscopic gastric biopsy revealed amyloid deposition in her stomach, indicating that her disease was complicated by amyloidosis (Figure 1(a)).

The patient had a regular tachycardia of 104 beats/min, an elevated blood pressure of 184/118 mmHg, and a normal temperature of 36.4°C. Cardiac auscultation revealed a third heart sound without an obvious murmur. Slight peripheral leg edema was observed, and the joints of her hands and feet were swollen and deformed. Neurological findings were normal. Laboratory findings are shown in Table 1. Her urinalysis showed mild proteinuria, microscopic hematuria, and an occasional granular cast. A complete blood count analysis showed leukocytosis and anemia, and a biochemical analysis showed hypoalbuminemia, renal dysfunction with an eGFR of 8.6 mL/min/1.73 m^2^, and a high B-type natriuretic peptide concentration of 3002.5 pg/mL. An immunological analysis revealed significant elevations in CRP and SAA levels; monoclonal immunoglobulins and free light chains were not detected in the patient's serum or urine.

**Figure 2:**
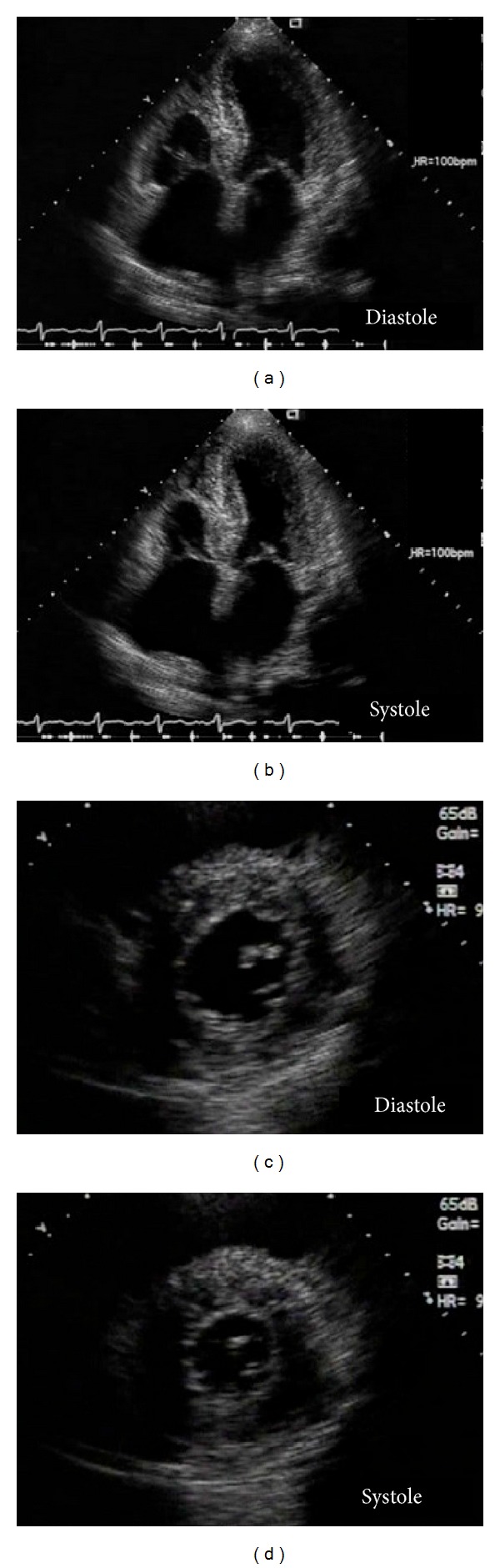
Transthoracic echocardiography 4-chamber (a and b) and short axis (c and d) views show concentric left ventricular (LV) hypertrophy, with a sparkling and granular myocardial texture; right ventricular hypertrophy; and bilateral atrial dilatation. The LV ejection fraction is preserved at 53%, and the mass significantly increased to 256.6 g.

**Figure 3:**
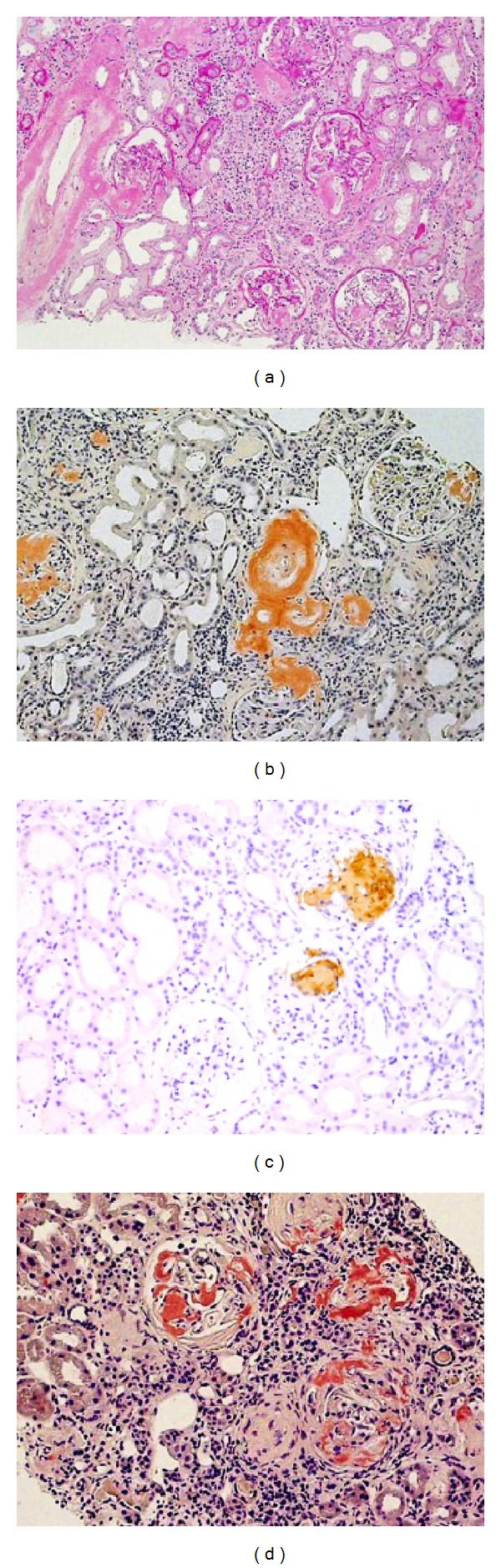
Renal biopsy. (a) Hematoxylin-eosin staining shows that approximately 40% of the glomeruli were globally sclerotic; however, the remaining glomeruli were minimally obliterated by mesangial matrix expansion, and homogeneous and eosinophilic deposits were evident along the capillary walls. Small arteries are thickened by eosinophilic deposits, and tubules are focally atrophic. (b) The deposits reveal positive reactions to Congo red. (c) Immunoperoxidase staining confirmed that the deposits included amyloid A protein. (d) After 4 years of tocilizumab treatments, Congo red staining revealed no regression of the amyloid deposition.

An ultrasound examination indicated diffusely enlarged thyroid glands, although thyroid function was normal. A chest X-ray scan showed cardiac enlargement, pulmonary congestion, and bilateral pleural effusion, and transthoracic echocardiography revealed concentric left ventricular (LV) hypertrophy with a sparkling and granular myocardial texture and right ventricular hypertrophy. The patient's LV ejection fraction and diastolic dimension were preserved at 53% and 46 mm, respectively; however, her left atrial diameter and septal and posterior wall thicknesses were significantly increased at 55 mm, 14.0 mm, and 14.7 mm, respectively. The LV mass had thus significantly increased to 256.6 g, which was determined according to Devereux's formula (Figure 2) [10]. Peak early and late diastolic LV inflow velocities could not be measured by transmitral Doppler imaging because of their fusion associated with the sinus tachycardia. No significant valvular disease was detected. We suspected that she had heart failure with preserved ejection fraction due to cardiac amyloidosis; however, we did not perform percutaneous endomyocardial biopsy owing to the patient's refusal and her progressive renal failure. A renal biopsy revealed that approximately 40% of her glomeruli were globally sclerotic. Small arteries were severely injured by amyloid A deposition, and the tubules were focally atrophic (Figures 3(a)–3(c)). These findings confirmed a diagnosis of AA amyloidosis involving the stomach and kidney, and she was suspected of having cardiac amyloidosis. We considered that her prognosis could be improved by eliminating the amyloid deposition in multiple organs.

Diuretics and a low dose of angiotensin converting enzyme inhibitor were added to her treatment regimen, and TCZ (8 mg/kg, repeated every 4 weeks) was administered, after receiving written informed consent. As a result, the patient's SAA and CRP levels were markedly decreased to within normal ranges after the first TCZ infusion, and her heart failure also gradually improved. However, 1 year after the TCZ treatments, hemodialysis was initiated because of worsening fluid retention and renal dysfunction.

**Figure 4:**
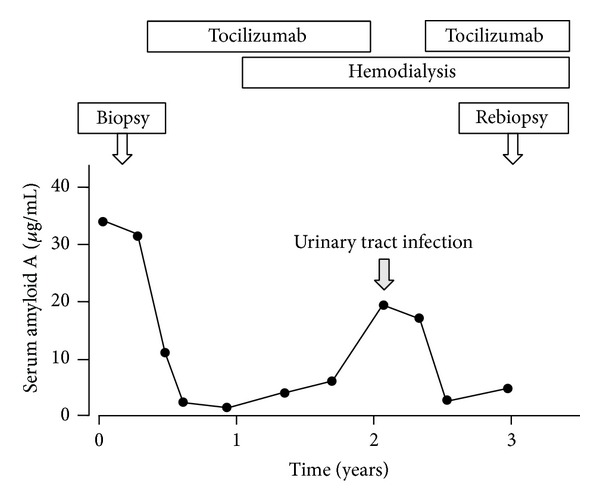
The clinical course and the changes of patient's serum amyloid A (SAA) levels. The tocilizumab treatment controlled her SAA levels, although there were transient SAA level elevations due to a urinary tract infection.

The TCZ treatment generally controlled the patient's SAA levels, although there were slight SAA level elevations when she temporarily discontinued TCZ treatment due to a urinary tract infection (Figure 4). She did not experience a relapse of heart failure, and her B-type natriuretic peptide concentration was maintained between 200 and 300 pg/mL.

A second endoscopic gastric biopsy, performed 3 years after the initiation of TCZ treatments, revealed the regression of the amyloid deposition (Figure 1(b)). A follow-up echocardiogram revealed that the patient's LV ejection fraction had increased to 62% and her left atrial dilatation had improved to 47 mm. The LV mass had minimally decreased to 230 g. We also performed a second renal biopsy to evaluate the possibility of withdrawing the patient from hemodialysis because her urinary volume was preserved at 300–500 mL/day, her gastric manifestations had significantly improved, and her heart failure remained well controlled. However, the second renal biopsy revealed that the amyloid deposition had not regressed and that her global glomerular sclerosis had progressed to approximately 70% of the glomeruli (Figure 3(d)). Currently, the patient is in good general condition.

## 3. Discussion

We report a patient with AA amyloidosis who was treated with TCZ. After treatment, her gastric manifestations improved significantly, but her renal amyloid deposition did not regress. This suggests two important points regarding TCZ treatment for AA amyloidosis. First, the therapeutic effects of TCZ can vary among different organs in patients with AA amyloidosis, as with the other disease modifying antirheumatic drugs (DMARDs) such as cyclophosphamide and tacrolimus [11]. Thus, a therapeutic benefit of TCZ in one involved organ should not be assumed to be similar in another organ; individual organ assessment is required. Second, TCZ treatments cannot always eliminate renal amyloid deposits in patients with AA amyloidosis. Recent case reports have revealed that TCZ treatments decrease proteinuria and improve or preserve renal function in patients with renal AA amyloidosis [8, 12–14]; however, the present patient's renal involvement was refractory to TCZ treatment.

We speculate that the following are three possibilities for the failure of TCZ to improve the present patient's renal symptoms. First, the removal efficiency of renal amyloid deposition may be strongly influenced by the renal damage severity. The present patient's eGFR was significantly decreased compared to that of patients in the previously mentioned case reports. Adequate treatment, including TCZ, is essential at an early stage for successful management of renal AA amyloidosis [13]. Second, the renal location of the amyloid deposition might be different in our patient and the previously reported patients. The clinical presentation and prognosis of renal AA amyloidosis were reportedly associated with whether the amyloid deposited predominantly in the glomeruli or in the vessels [15, 16]. Moreover, therapeutic response of DMARDs may vary among different renal cell types in patients with AA amyloidosis. Falck et al. first demonstrated that renal amyloid deposits were resolved in a case with renal AA amyloidosis during 2-year treatment with cyclophosphamide, using light, electron, and immunofluorescence microscopy [17]. They described that the mesangial amyloid substance is degraded to granular material and that the subepithelial amyloid deposits are resolved by mechanisms similar to those involved in the resolution of subepithelial immune complex deposits. Finally, the regression of renal amyloid deposition may require long-term treatment with TCZ because tissue turnover is considerably slower in the kidney than in the gastric mucosa. Further investigation in patients with renal AA amyloidosis is required to determine the therapeutic effects of TCZ.

Cardiac deposition is rare in patients with AA amyloidosis, and this presentation is associated with a poor prognosis [18, 19]. We suspected that the present patient had cardiac amyloidosis on the basis of the presence of LV hypertrophy with granular-sparkling myocardial texture, in the absence of other cardiac or systemic diseases [20]. However, we could not make a definite diagnosis because of the absence of histologic confirmation. From the present case, conclusions cannot be drawn regarding the therapeutic effects of TCZ on cardiac amyloidosis. Davis et al. reported that RA patients frequently have preserved ejection fraction at the first diagnosis of heart failure and that their prognosis following onset of heart failure is poor compared with non-RA patients [21]. Thus, our patient should be carefully followed up, regardless of whether she has cardiac amyloidosis.

In conclusion this experience suggests that, in patients with AA amyloidosis, the therapeutic effects of TCZ can differ in different organs. Thus, each involved organ should be individually assessed for therapeutic effect.

## Figures and Tables

**Table 1:** Laboratory findings on admission.

Urinalysis		
Protein	1+	
Occult blood	1+	
Glucose	−	

Urine sediment		
WBC	1–4	/HPF
RBC	1–4	/HPF
Granular casts	1+	/LPF

Complete blood counts		
WBC	13600	/mm^3^
RBC	355	×10^4^/mm^3^
Hemoglobin	9.6	g/dL
Hematocrit	29.9	%
Platelets	81.9	×10^4^/mm^3^

Biochemistry		
CRP	3.4	mg/dL
SAA	32.3	*μ*g/mL
Total protein	5.8	g/dL
Albumin	3.2	g/dL
Glucose	94	mg/dL
Uric acid	9.6	mg/dL
BUN	55	mg/dL
Scr	4.35	mg/dL
eGFR	8.6	mL/min/1.73 m^2^
Sodium	139	mEq/L
Potassium	4.8	mEq/L
Chloride	106	mEq/L
Serum *β*2-MG	11.4	mg/L
Urinary *β*2-MG	4189	*μ*g/L

Immunology		
RF	3.4	IU/mL
ANA	−	
Anti-CCP Ab	3.4	U/mL
MMP3	322	ng/mL
Complement	37	U/mL
C3	62.4	mg/dL
C4	20.6	mg/dL
IgA	269.2 2	mg/dL
IgG	1119.9	mg/dL
IgM	76.8	mg/dL

Endocrinology		
HbA1c	6.2	%
BNP	3002.5	pg/mL

RBC: red blood cell; WBC: white blood cell; HPF: high-power field; CRP: C-reactive protein; SAA: serum amyloid A protein; BUN: blood urea nitrogen; Scr: serum creatinine; eGFR: estimated glomerular filtration ratio; *β*2 MG: *β*2-microglobulin; HbA1c: haemoglobin A1c; BNP: B-type natriuretic peptide; RF: rheumatoid factor; ANA: antinuclear antibody; anti-CCP Ab: antibodies against cyclic citrullinated peptide; MMP3: matrix metalloproteinase 3; Ig: immunoglobulin.
